# The anesthesia workforce in Canada: a methodology to identify physician anesthesia providers using health administrative data

**DOI:** 10.1186/s12960-023-00820-w

**Published:** 2023-04-26

**Authors:** Sarah Simkin, Beverley A. Orser, C. Ruth Wilson, Ivy Lynn Bourgeault

**Affiliations:** 1 Canadian Health Workforce Network and Faculty of Medicine, University of Ottawa, 600 Peter Morand Crescent, Ottawa, ON K1G 5Z3 Canada; 2 Department of Anesthesiology & Pain Medicine, University of Toronto, 12th Floor, 123 Edward Street, Toronto, ON M5G 1E2 Canada; 3 Department of Anesthesia, Sunnybrook Health Sciences Centre, 2075 Bayview Ave., M-wing, 3rd floor, Room M3200, Toronto, ON M4N 3M5 Canada; 4 Professor Emerita, Department of Family Medicine, Queen’s University, 220 Bagot Street, Kingston, ON K7L 3G2 Canada; 5 Canadian Health Workforce Network and School of Sociological and Anthropological Studies, University of Ottawa, 120 University Private, Ottawa, ON K1N 6N5 Canada

**Keywords:** Anesthesia services, Anesthesiology, Health workforce, Delivery of health care

## Abstract

**Background:**

Safe and timely anesthesia services are an integral component of modern health care systems. There are, however, increasing concerns about the availability of anesthesia services in Canada. Thus, a comprehensive approach to assess the capacity of the anesthesia workforce to provide service is a critical need. Data regarding the anesthesia services provided by specialists and family physicians are available through the Canadian Institute for Health Information (CIHI) but collating the data across delivery jurisdictions has proven challenging. As a result, information related to the activity of physician anesthesia providers is routinely excluded from annual physician workforce reports. Our goal was to develop a novel approach to identifying and characterizing the anesthesia workforce on a pan-Canadian scale.

**Methods:**

The study was approved by the University of Ottawa Office of Research Ethics and Integrity. We developed a methodology to identify physicians who provided anesthesia services in Canada between 1996 and 2018 using data elements from the CIHI National Physician Database. We iteratively consulted with expert advisors and compared the results with Scott’s Medical Database, the Canadian Medical Association (CMA) Masterfile, and the College of Family Physicians of Canada membership database.

**Results:**

The methodology identified providers of anesthesia services using data elements from the CIHI National Physician Database, including categories of the National Grouping System, specialty designations, activity levels and participation thresholds. Physicians who provided anesthesia services only sporadically and medical residents-in-training were excluded. This methodology produced estimates of anesthesia providers that aligned with other sources. The process we followed was sequential, transparent, and intuitive, and was strengthened by collaboration and iterative consultation with experts and stakeholders.

**Conclusions:**

Using physician activity patterns, this novel methodology allows stakeholders to identify which physician provide anesthesia services in Canada. It is an essential step in developing a pan-Canadian anesthesia workforce strategy that can be used to examine patterns and trends related to the workforce and support evidence-informed workforce decision-making. It also establishes a foundation for assessing the effectiveness of a variety of interventions aimed at optimizing physician anesthesia services in Canada.

## Background

Anesthesia providers are key supporters of comprehensive surgical, obstetrical and critical care, emergency medicine, and pain management programs. The World Health Organization recognizes the importance of anesthesia services in the provision of safe surgical care in the operating room and beyond, including emergency airway management and resuscitation in trauma and obstetrics [1]. International standards[2] and programs and tools[3, 4] that include assessments of anesthesia capacity[5, 6] have been developed to support access to safe anesthesia care.

In some Canadian regions, access to timely anesthesia care has been limited [7]. Multiple issues are at play, including increasing demand for anesthesia services both inside and outside operating rooms [8, 9], constrained availability of anesthesia care providers and other resources[10] (e.g., a lack of anesthesia assistants), geographic and workforce distribution challenges [12], and an aging workforce. Timely access to care is of particular concern for Indigenous and non-Indigenous peoples living in rural and remote regions of the country [11, 12]. Despite the importance of evidence-informed approaches to workforce decision-making, pan-Canadian data that capture the need for anesthesia care, the number of anesthesia providers and the volume of clinical service they provide, and the gap between population demand and workforce capacity, are conspicuously absent.

In Canada, anesthesia care is provided by physicians who fall into several major groups. Most is provided by specialty-trained anesthesiologists who have passed a national examination established by the Royal College of Physicians and Surgeons of Canada. Some care, often in rural or remote regions, is provided by family physician anesthetists who have undertaken enhanced skills training and may have a Certificate of Added Competence issued by the College of Family Physicians of Canada. Other physicians who work in emergency departments also provide certain anesthesia services (such as conscious sedation for reduction of fractures). In some settings, physician anesthesia providers are supported by anesthesia assistants under a delegated medical act framework. This study focuses on physician anesthesia providers.

The sufficiency of the anesthesia workforce in Canada is difficult to assess. Provider-to-population ratios suggest that Canada’s density of anesthesia providers (12.42 per 100 000 population) is lower than that of many other countries (including the United States (20.82 per 100 000), the United Kingdom (17.85 per 100 000), and Australia (23.09 per 100 000)) [13]. However, without accounting for differential population needs and the characteristics of physicians impacting their capacity to provide service—such as age and gender, skill mix, distribution, and scope of practice—an assessment of sufficiency is nearly impossible. Reports of shortages of anesthesia providers [12] and pressure to address pandemic-related surgical backlogs are intensifying the imperative to better understand this workforce.

Issues related to planning for the Canadian anesthesia workforce have been explored previously [14–17]. Planning efforts have used registration data [18] and surveys of health care facilities [19] to anchor estimates of the stock of physician anesthesia providers. However, a proactive and prospective approach to workforce planning for this workforce has yet to be adopted by leaders and stakeholders [18]. This is likely, in part, because access to comprehensive and high-quality data at a pan-Canadian scale remains a barrier to planning. ‘Head counts’ based on surveys and analyses of provincial and territorial physician registries and professional association membership databases have significant drawbacks. In particular they are neither standardized nor linkable and they often fail to capture the types and levels of service output provided by individual physicians within the workforce. Thus, they may not represent a sufficiently robust foundation for workforce modeling on a pan-Canadian scale. Furthermore, because family physician anesthetists have not been consistently captured by any of these data collection strategies, an essential cadre of providers that are delivering important care at the community level is missing from workforce and policy analyses.

Clinical activity data available through the Canadian Institute for Health Information (CIHI) represent a potentially robust source of workforce data. Data originating in provincial and territorial medical systems and collected by CIHI are screened, standardized, and collated in accordance with rigorous standards to maintain high-quality information. A variety of billing schemes for anesthesia services are in place across the country, including time-based billing, flat-rate billing for certain services (such as consultations and procedures), and escalating scales, resulting in data that are disparate and difficult to harmonize. As a result, data concerning the activity of anesthesia providers have been routinely suppressed in CIHI reports due to challenges related to the comparability of service counts across provincial and territorial jurisdictions. Despite their complexities and limitations, data from CIHI have important features that are missing from other data sources: standardized and high-quality pan-Canadian information (including the type and volume of service provided) for all physician anesthesia providers.

This study responds to an opportunity to leverage the specific strengths of CIHI data to create a solid foundation for pan-Canadian anesthesia workforce projections and modeling, and to provide support for evidence-informed workforce policy and planning. The specific objective of this study was to develop a methodology to identify physicians providing anesthesia care using data from the CIHI National Physician Database. By taking into account physician activity patterns, we aim to generate accurate estimates of the number of anesthesia care providers in Canada that can be used as a starting point for the development of a comprehensive pan-Canadian anesthesia workforce strategy.

## Methods

Approval for the study was granted by the University of Ottawa Office of Research Ethics and Integrity (S-01-21-6385).

The CIHI National Physician Database contains information on the payments and clinical service activities of physicians in Canada. Data come from provincial and territorial health systems and are categorized using a standardized set of data elements. The CIHI National Grouping System (NGS) [19] organizes fee codes from each province and territory into homogenous categories, allowing for a comparison of physician services and activities across the country. We used two NGS categories—NGS 074, which captures ‘Nerve Block’ services and NGS 075, which captures all ‘Other Anesthesia’ services—as a starting point for identifying physicians providing anesthesia services. (For example, the billing codes for anesthesia for cholecystectomy in Ontario (S287C), Alberta (63.14) and New Brunswick (1140 2) all map to the same NGS category (075) and the service is characterized as ‘Other Anesthesia.’) However, these two categories are very broad, with thousands of codes and services mapping onto them, so additional inclusion and exclusion criteria were necessary to delineate whether physicians providing ‘Nerve Block’ or ‘Other Anesthesia’ services could be characterized as belonging to the anesthesia workforce.

The initial step in the development of the methodology was to capture all data associated with any physician who provided anesthesia services in Canada. We focused on 10 provincial and territorial jurisdictions: Alberta, British Columbia, Manitoba, New Brunswick, Newfoundland, Nova Scotia, Ontario, Prince Edward Island, Saskatchewan, and Yukon. Data from Québec were not included or analyzed because billing data relating to physicians associated with the Régie de l’assurance maladie du Québec may not be used for any purpose other than the specific analyses produced by CIHI. Two of the northern territories—The Northwest Territories and Nunavut—did not submit insurance data to CIHI.

Although historical data in the National Physician Database extend back to 1989, the first year for which reliable data were available for all jurisdictions (ten provinces and one territory) was 1996. Prior to 1996, some jurisdictions used alternate fee code structures and including those years would generate comparability issues and preclude trend analysis. The most recent year for which complete data were available at the time the data were requested was 2018. The resultant dataset spans 22 years, from 1996 to 2018.

From the National Physician Database, CIHI analysts generated a list of physicians who were designated as having an ‘Anesthesia’ specialty as well as those physicians who provided at least one Nerve Block or Other Anesthesia service between 1996 and 2018. The dataset included a unique physician identifier, the binary sex and birth year of the physician, the year the physician graduated from medical school, and the location of the physician’s undergraduate medical training. Other data elements captured included physician specialty, geography (urban or rural), National Grouping System codes, number of services, and payments (adjusted amounts paid for each service, total payments, and payments through fee-for-service, alternative payment programs, group payments, and aggregate payments) for each fiscal period. In addition, a numerical measure of how similar each physician’s practice is to the average practice of physicians with ‘Anesthesia’ specialty was included in the dataset.

Nearly 38 000 physicians met the initial inclusion criteria. Since we knew that this estimate far exceeded the true size of the anesthesia workforce, a methodology to identify who actually contributed to the anesthesia workforce was necessary. We iteratively explored options for delimiting the workforce to better capture only physicians who should be included in the dataset. We focused primarily on minimizing the likelihood of a Type I error (i.e., missing physicians who should be included in the workforce), while fully acknowledging that a certain probability of Type II error (i.e., inappropriately including physicians in the anesthesia workforce) was unavoidable. We used the absolute number and proportion of physicians with specialties other than ‘Anesthesia’ or ‘Family Practice’ captured and included in the workforce to guide our assessment of the degree of Type I and Type II error. Methodological options resulting in the inclusion of large numbers of these physicians indicated an unacceptable Type II error, while the complete exclusion of physicians with alternative specialties indicated an unacceptable Type I error.

We explored establishing inclusion and exclusion criteria based on physician specialty, participation (NGS categories), and the level of clinical service provision. We established inclusion criteria based on a series of activity thresholds related to absolute payments for anesthesia services (‘Other Anesthesia’ and ‘Nerve Block’ services alone or in combination), the percentage of total income represented by anesthesia activities (more than 1% for ‘Other Anesthesia’, ‘Nerve Block’ services, or both), and the number of services provided (more than 1, 5, 8, or 10 ‘Nerve Block’ services). We iteratively explored the impact of each of these thresholds on the size and specialty composition of the anesthesia workforce.

The results of each exploratory iteration were validated by comparing the results of each provisional method with other available data related to anesthesia providers in Canada. Because data from disparate data sources are not standardized or linkable, record-by-record comparisons were impossible; comparisons of the aggregate number of providers in each year of the study period were made.

We compared the number of anesthesia providers generated by our methodology to the number of anesthesia specialists identified in Scott’s Medical Database [20] in each year of the study period. Scott’s Medical Database includes physicians who have an MD degree and a valid mailing address and who are active in clinical and non-clinical practice, but excludes medical residents-in-training, physicians working in the military, semi-retired and retired physicians, and physicians who requested that their information not be published.

We also compared our results to the number of anesthesiology specialists listed in the Canadian Medical Association (CMA) Masterfile [21] for the period between 2000 and 2018. The Masterfile includes part-time and semi-retired physicians as well as non-clinicians and physicians who maintain a license to practice but work primarily in administrative positions, but excludes medical residents-in-training as well as physicians who are over the age of 80 years.

Finally, we compared our estimates of the number of family physicians providing anesthesia services to the number in the College of Family Physicians of Canada membership database [22]. This data source captures physicians with a specific Certificate of Added Competence in Family Practice Anesthesia but not family physicians who practice anesthesia without this designation.

In addition to comparing the results generated by the application of our methodology with extant anesthesia workforce data, we undertook consultation meetings with advisory groups that were composed of leaders within the academic and community anesthesia communities (Appendix). These stakeholders universally recognized the importance of grounding planning and policy in high-quality data. The consultations served as a forum to discuss priorities (such as capturing all physician anesthesia providers), potential options (such as using data sources that permit assessment of clinical activity rather than only specialty certifications or self-report as a basis for inclusion in the workforce), and data-related issues and challenges. Throughout the process of developing this methodology, we received guidance from these advisors as to the acceptability of the methodology and the face validity of the results.

The final methodology represents a process that begins inclusively and sequentially excludes physicians based on participation and service volume thresholds. The methodology captured physicians from multiple specialty groups, minimized the number of physicians who were inappropriately excluded from the workforce, and resulted in workforce numbers that are comparable to estimates from other sources.

## Results

**Fig. 1:**
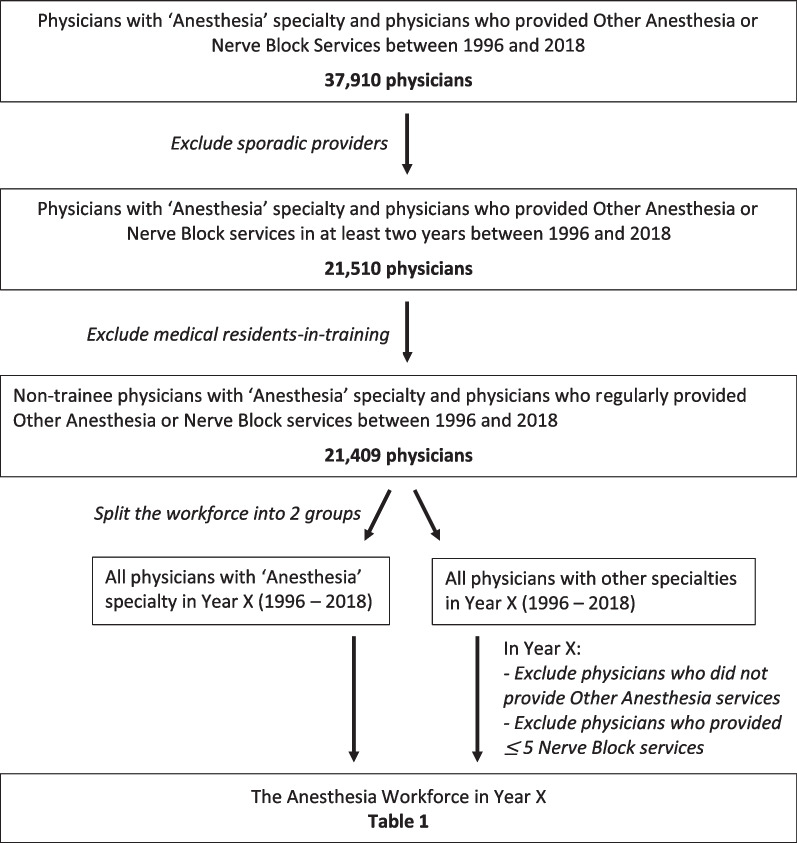
Overview of methodology

The methodology we developed started with all physicians in the National Physician Database with ‘Anesthesia’ specialty and all physicians who provided ‘Other Anesthesia’ or ‘Nerve Block’ services between 1996 and 2018 (Fig. 1). In our dataset, 37 910 physicians met the initial inclusion criteria.

Many physicians who were captured in this initial dataset were likely involved only in sporadic provision of a service that was categorized as ‘Other Anesthesia’ or ‘Nerve Block’. We presume that this group includes surgeons and other physicians who provided nerve blocks or light sedation while delivering surgical or other services. Given that our goal was to identify physicians who provide anesthesia services on a regular basis, physicians who provided service for a period of only one or two years (between 1996 and 2018) were excluded. The exception to this exclusion criterion was physicians who appeared in the dataset in only 1996 or 1997, or in only 2017 or 2018. This exception ensured that physicians who were ending their careers in 1996 or 1997, or who were beginning their careers in 2017 or 2018, were not excluded inappropriately. After sporadic providers were removed, 21 510 physicians remained in the dataset.

In some provinces, medical residents-in-training are allowed to ‘moonlight’ and provide anesthesia services while still in training programs. Given that the focus of this study is on non-trainee physicians, we opted to exclude physicians with specialties characterized as ‘Resident’ throughout the study period. However, physicians who provided anesthesia services in one year with a ‘Resident’ specialty and subsequently provided service with an ‘Anesthesia’ specialty are retained in the dataset. After removing 101 trainee physicians, 21 409 physicians remained, as shown in Fig. 1.

Many of the physicians included in the initial dataset had a specialty other than ‘Anesthesia.’ In fact, 34 specialties in addition to ‘Anesthesia’ were represented in this dataset.

The next step in the methodology was to exclude physicians with other specialties based on their activity patterns. We iteratively tested a series of activity thresholds before settling on the following approach: To capture physicians who routinely provide anesthesia services—rather than those who provided occasional nerve blocks during their non-anesthesia specialty clinical practice—we first stipulated that the physician provide at least one ‘Other Anesthesia’ service in any given fiscal year. We also required that physicians provided more than five ‘Nerve Block’ services in that same year to be retained in the dataset. Once these activity level criteria are applied, the number of specialties (with more than five providers) in addition to ‘Anesthesia’ represented in the workforce in any given year ranged from four to seven.

Activity thresholds are not applied to physicians with ‘Anesthesia’ specialty; these physicians are included in the workforce regardless of their activity patterns.

The overall results of the application of the methodology are shown in Table 1. In each year of the study period, we estimated the number of physicians participating in the anesthesia workforce. Estimates of the total number of physicians in Canada and the stock of anesthesia providers from other sources that were used as comparators, including the Scott’s Medical Database and the Canadian Medical Association Masterfile, as well as the College of Family Physicians of Canada membership database, are also shown in Table 1.

**Table 1:** The Physician Workforce and Anesthesia Providers (1996–2018): Estimates of provider stock from different data sources

	1996	1997	1998	1999	2000	2001	2002	2003	2004	2005	2006	2007	2008	2009	2010	2011	2012	2013	2014	2015	2016	2017	2018
Anesthesia Workforce: Current Study*	2016	2039	2078	2160	2223	2246	2281	2357	2438	2535	2623	2723	2840	3035	3111	3230	3318	3405	3488	3560	3622	3649	3681
**Anesthesia specialists**																							
Current study*	1672	1681	1694	1721	1769	1822	1849	1922	1990	2038	2119	2207	2292	2480	2544	2617	2672	2731	2803	2850	2905	2949	3020
SMDB*	1682	1678	1715	1761	1800	1834	1840	1852	1874	1895	1909	2013	2070	2184	2262	2345	2375	2472	2504	2576	,657	2716	2834
CMA Masterfile**					1738	1763	1799	1827	1852	1892	1979	2038	2094	2173	2212	2284	2360	2444	2474	2500	2513	2569	2573
**Family practice anesthesia**																							
Current Study*	300	314	342	363	384	366	370	383	397	430	425	441	461	469	478	507	525	544	561	575	577	564	534
CFPC membership database																				206	307	328	348
**All Physicians**																							
SMDB**	39 686	39 901	40 691	41 332	42 033	42 680	43 612	43 936	44 467	45 268	45 774	46 900	48 383	50 671	51 902	54 033	56 152	58 312	60 199	62 143	63 793	65 735	68 894
CMA masterfile**					41 717	42 594	43 422	44 924	45 385	45 484	46 625	47 705	49 397	50 477	52 123	52 972	54 914	56 708	57 232	59 121	60 194	62 905	63 577

*Includes physicians from Alberta, British Columbia, Manitoba, New Brunswick, Newfoundland, Nova Scotia, Ontario, Prince Edward Island, Saskatchewan, and Yukon

**Includes physicians from Alberta, British Columbia, Manitoba, New Brunswick, Newfoundland, Nova Scotia, Ontario, Prince Edward Island, Saskatchewan, and the Territories

When all physicians were grouped together in a longitudinal dataset (using the unique physician identifier assigned by CIHI to ensure that physicians were captured only once), 6774 individual physicians were characterized as belonging to the anesthesia workforce between 1996 and 2018; annual estimates of the number of physicians participating in the workforce ranged from around 2000 in 1996 to more than 3600 in 2018. These estimates are comparable to estimates from other available data sources and they have good face validity.

## Discussion

In response to an urgent need to better understand who is delivering anesthesia services in Canada, we developed a sequential and transparent methodology that used pan-Canadian physician activity data to identify anesthesia providers. Between 1996 and 2018, the number of physicians identified by our methodology as providing anesthesia services increased 1.8-fold, from 2016 to 3681 physicians. Over the same period, the total physician workforce increased 1.7-fold. Our methodology generated workforce numbers that are comparable to estimates of workforce stock from other sources, particularly in the earlier years of the study period. In more recent years, the estimated number of anesthesia providers according to our methodology and estimates available from Scott’s Medical Database and the Canadian Medical Association Masterfile diverged more, likely due to the different purposes of the respective data sources and the inclusion and exclusion criteria for each dataset.

Our methodology was designed with the ultimate purpose of facilitating workforce planning, and thus was activity-based and included physicians of all ages and specialties. The Scott’s Medical Database is a marketing directory that includes physicians in both clinical and non-clinical practices (such as those involved in leadership and administrative work) and excludes any physician who requests that their information not be published, whereas the Masterfile excludes physicians over age 80 but includes non-clinicians such as physicians working in administrative fields. Increasing specialty stratification that has taken place in the Canadian Medical Association Masterfile (hence, providers classified as specializing in ‘Pain Medicine’ or ‘Critical Care Medicine’ are not included in counts of ‘Anesthesiology’ even though they may delivery anesthesia services) likely accounts for some of the difference between the estimates, and highlights that acknowledgement and careful consideration of the factors contributing to the degree of uncertainty associated with workforce estimates from any source is necessary.

This study’s main strength is the use of high-quality pan-Canadian data, including clinical activity thresholds, to identify all members of the anesthesia workforce. Harmonization of clinical activity data through the NGS is an important facilitator of pan-Canadian workforce analyses and the addition of clinical activity thresholds to the process for identifying physicians as belonging to the workforce uniquely characterizes our approach. We are confident that we captured almost all physicians who delivered anesthesia services in these Canadian jurisdictions, including anesthesia specialists, family physician anesthesia providers, and physicians from other specialties with overlapping skillsets. The ability to identify all groups of providers is particularly important for understanding sufficiency of the workforce more broadly, for characterizing the state of anesthesia service delivery in rural and remote health systems across the country, and for developing potential solutions to maldistribution.

Engagement with stakeholders is recognized as a leading practice in health workforce planning [23] and stakeholders played an essential role in the development of the methodology presented here. The names of those involved in the Working Group, which regularly provided advice, are listed in the Appendix. The involvement in this project of representatives from the Association of Canadian University Departments of Anesthesia, the Canadian Anesthesiologists’ Society, the College of Family Physicians of Canada, and the Society of Rural Physicians of Canada, as well as other clinical leaders, highlights the interest in, and importance of, this workforce assessment and planning project. This work has created a forum for collaboration on issues related to the provision of anesthesia services, including the education and training pathway, support for physicians in clinical practice, and advocacy for system-level transformations.

Our work had some important limitations. The dataset was missing most anesthesia providers in Quebec, the Northwest Territories, and Nunavut. The methodology also missed small numbers of providers who were remunerated outside of provincial billing structures and physicians with specialties other than ‘Anesthesia’ who made only a few nerve block payment claims each year. Data from Yukon were missing (for the time period 2006–2012), and there were some inconsistencies in mapping of some clinical services to NGS categories. A second important limitation is that the most recent available data are now nearly five years old. Ongoing improvements to health workforce data infrastructure at the provincial and territorial level, as well as at CIHI, are needed to make the collected information accessible in a timely fashion and thus more useful for planning.

Having established a methodology to identify physicians who belong to the anesthesia workforce, we are now in a position to explore other important questions related to the characteristics and activity patterns of anesthesia providers as well as workforce trends: who provides anesthesia services and how much; how are activity and participation rates changing over time; and the rates of attrition and retirement from the anesthesia workforce. We can also address additional questions related to the age and gender structure of the workforce, the numbers of women and international medical graduates in anesthesia, the family practice anesthesia workforce, the rural anesthesia workforce, and workforce mobility and flow.

Our experiences have demonstrated that it can be difficult to confidently characterize a workforce when clinical activities cut across specialty providers, such as is the case with surgical and obstetrical services. Although the methodology that we developed is highly specific to the physician anesthesia workforce, our approach can provide a roadmap for workforces with similar challenges. The principles underlying these analyses are transferrable to other groups of physicians, other sectors, and other countries and jurisdictions. Integrating head counts and clinical activity data, and engagement with expert advisors to assess and validate different methods, inclusion and exclusion thresholds, and results and outputs, offer important benefits to health systems more broadly.

Strong efforts have been made in the past to model the future Canadian anesthesia workforce [16, 18, 19]. However, the collaborative approach to planning called for by Byrick and colleagues in 1999 [24] and again in 2016 [20], along with iterative evaluation, validation, and revision of these models, remains elusive. In the meantime, while many of the planning principles acknowledged and embraced in these planning efforts remain essential and relevant, leading practices in planning have evolved. Utilization and demand-based metrics are recognized as insufficient in elaborating estimates of need. More sophisticated health workforce data infrastructure both allows for and requires a more robust approach to planning [25]. High-quality pan-Canadian workforce data are necessary for future anesthesia workforce planning efforts, and this study establishes a foundation that facilitates using CIHI data for planning. In the future, the linking together of other datasets housed at CIHI offers a potentially powerful opportunity to understand the patient populations served by anesthesia providers, including the types of care they receive and their outcomes. While true pan-Canadian population needs-based planning and outcomes assessment for the anesthesia workforce remain aspirational, the mobilization of CIHI data related to this workforce and the development of this methodology represent small steps forward.

## Conclusions

Identifying and characterizing the workforce is a foundational component of effective workforce planning. The methodology presented in this report represents a critical step in accurately identifying all the members of the anesthesia workforce in Canada. In developing this methodology, we have adopted leading practices to accomplish our goal. This methodology is a pragmatic approach that leverages the unique strengths of CIHI data (high-quality pan-Canadian information, including all providers of anesthesia services, identified on the basis of their clinical activity rather than by specialty or self-report). It will support the development of a national workforce strategy and provide a foundation for assessing the impact of interventions aimed at improving access to anesthesia care.

## Data Availability

The data that support the findings of this study are available from the Canadian Institute for Health Information but restrictions apply to the availability of these data, which were used under license for the current study, and so are not publicly available.

## Appendix

### Working group members

**Table:**

Advisor	Affiliation
Ramiro Arellano	Queen’s University
Daniel Bainbridge	Western University
Michael Bautista	Memorial University
Pierre Beaulieu	University of Montreal
Janice Chisholm	Dalhousie University
Chris Christodoulou	University of Manitoba
Derek Dillane	University of Alberta
Lucie Filteau	University of Ottawa
Paul Gill	University of Toronto; Western University
Gregory M. T. Hare	University of Toronto
Vaibhav Kamble	University of Toronto
Brent Kennedy	Northern Ontario School of Medicine University
Colin McCartney	University of Toronto (formerly University of Ottawa)
Dolores McKeen	Memorial University
Jason McVicar	University of Ottawa
Victor Ng	Western University
Joel Parlow	Queen’s University
James Paul	McMaster University
Roanne Preston	University of British Columbia
Mateen Raazi	University of Saskatchewan
Thomas Schricker	McGill University
Erich van der Linde	University of Calgary; Society of Rural Physicians of Canada
Homer Yang	Western University

## Abbreviations

CMA: Canadian Medical Association
CIHI: Canadian Institute for Health Information
NGS: National Grouping System
